# Modeling and Simulation of Control Actuation System with Fuzzy-PID Logic Controlled Brushless Motor Drives for Missiles Glider Applications

**DOI:** 10.1155/2015/723298

**Published:** 2015-11-03

**Authors:** Murali Muniraj, Ramaswamy Arulmozhiyal

**Affiliations:** Department of Electrical and Electronics Engineering, Sona College of Technology, Salem 636005, India

## Abstract

A control actuation system has been used extensively in automotive, aerospace, and defense applications. The major challenges in modeling control actuation system are rise time, maximum peak to peak overshoot, and response to nonlinear system with percentage error. This paper addresses the challenges in modeling and real time implementation of control actuation system for missiles glider applications. As an alternative fuzzy-PID controller is proposed in BLDC motor drive followed by linkage mechanism to actuate fins in missiles and gliders. The proposed system will realize better rise time and less overshoot while operating in extreme nonlinear dynamic system conditions. A mathematical model of BLDC motor is derived in state space form. The complete control actuation system is modeled in MATLAB/Simulink environment and verified by performing simulation studies. A real time prototype of the control actuation is developed with dSPACE-1104 hardware controller and a detailed analysis is carried out to confirm the viability of the proposed system.

## 1. Introduction

Brushless DC motor drive is used extensively in process industries, robotics, aerospace, and home appliance. In defense gliders and missiles are used to assail rivals. A motion controller is realized to control fins of missiles and gliders to reach their target for which a BLDC motor drive is used to control fins. BLDC motor has features of protracted performing viability, high dynamic response, and efficiency. BLDC motor response is the challenge task to control position fins in desired direction with reference to the steering command. BLDC motor offers efficient speed torque characteristics and closed loop control techniques which is a cause to use motor in motion control applications [1]. Nasri et al. presented the mathematical model construction of a brushless DC motor via MATLAB/Simulink [2] to view real time performance of motor in nonlinear conditions. A power electronics switching inverter is used to drive BLDC motor for control actuation system. The PWM-generation logic is to switch inverter to drive BLDC motor depending on error generator from controller. To acquire feedback signals a quadrature encoder model is used to acquire rotor position information of BLDC motor to controller [3, 4]. Modeling of controller is primary task of actuation system to maintain optimum response various command inputs. The conventional PID controller is used in many industries even though delivery of position response is poor for nonlinear type of system [5, 6]. The conventional PID control had been implemented in BLDC motor drive. But these controllers suffer from drawbacks: lack of performance in nonlinear system and more rise time with oscillatory response [7–9].

Recently, as alternative to PID controller, a fuzzy based control technique is considered for BLDC motor drive to optimize gain values in systematic approach [10–12]. The proposed fuzzy technique includes self-tuning response to optimize gain values of controller for different command inputs.

Fuzzy logic inference system has human intelligence in nature and is associated with rule based system successfully applied in control applications [13]. The following are virtues of fuzzy control: (i) improved stability, (ii) less sensitivity to load dynamics, (iii) simple control configuration, and (iv) low cost and more time.

Ko explained application of fuzzy logic PI controller in controlling shaft position of a motor [12]. Todić et al. [8] implemented PID control techniques in BLDC motor for electromechanical actuation applications. The servo-based actuators are designed to control fins surfaces through planetary gear system and linkage mechanism such as wings and fins for aerodynamic control and its steering. The FCAS controls four actuators and associated control drive system with integrated electronics into a ring that matches the mold outer line of the missile. The main objective of this paper is to model fin control actuation system using fuzzy-PID controller in missiles glider applications. The complete system is modeled with MATLAB/Simulink environment. The proposed system is validated through a real time test bench dSPACE controller cp1104.

## 2. Modeling of BLDC Motor Drive

**Figure 1:**
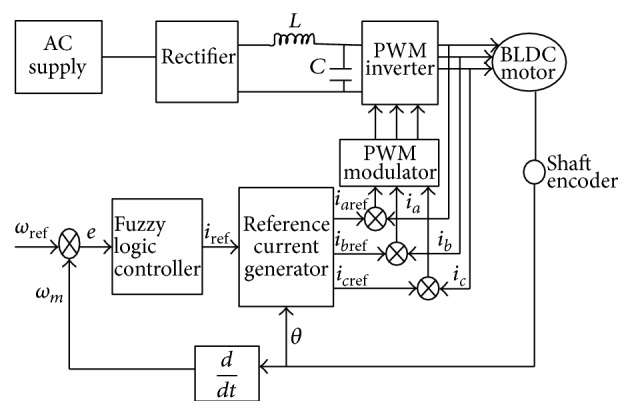
Block diagram of proposed setup.

The proposed system BLDC motor drive for control actuation system with fuzzy-PID control is modeled using MATLAB/Simulink and overview of blocks is shown in Figure 1.

### 2.1. BLDC Motor Modeling

BLDC motor terminal voltage equation can be represented in (1) and derived as state space model equations and simulated in MATLAB toolbox [14]:

(1) Va=RaIa+Ladiadt+Macdibdt+Mbcdicdt+ea,Vb=RbIb+Lbdibdt+Macdiadt+Mbcdicdt+eb,Vc=RcIc+Lcdicdt+Macdibdt+Mbcdiadt+ec,

where *R*_*a*-*b*_ is resistance per phase, equal to all phases. *L*_*a*-*b*_ is inductance per phase equal to all phases. *M*_*ac*_ and *M*_*bc*_ are mutual inductance. For BLDC motor net effect value will be Zero. *i*_*a*_, *i*_*b*_, and *i*_*c*_ are stator current/phase. *V*_*a*_, *V*_*b*_, and *V*_*c*_ are the phase voltage of the winding.

Motor parameters torque and Electromagnetic Force (EMF) of BLDC motor in trapezoidal nature are calculated in (2) in which *C*_*o*_ and *C*_*v*_ are friction torque in static and dynamic conditions and *T*_*l*_ is load torque of the motor [15–17]:

(2) ea=faθKeω,eb=fbθKeω,ec=fcθKeω,Te=Tl−Co−Cv.

The Final Output power is developed by motor

(3) $$\begin{array}{c} P=Te*\omega , \end{array}$$

where *ω* is Angular Velocity of the motor in radians per second and *P* is total power output.

**Figure 2:**
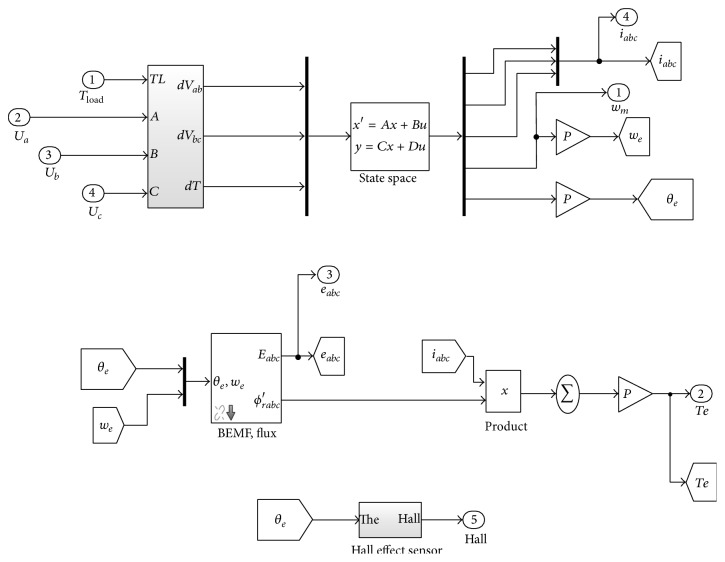
Mathematical model of BLDC motor in state space equation.

The motor parameter such as stator resistance, inductance, and back EMF constant parameter implicates control response of motor. The motor parameters are responsible for maximum overshoot, unsteady state with more transient response which reduces time response of control actuation system. To overcome the above drawbacks motor gain parameters need to be tuned using a conventional PID controller which is not self-tuned and compactable for time varying system. For better dynamic response a fuzzy-PID controller is proposed to optimize gain parameters as a self-tuned system which is diverse with input command signal. The BLDC motor parameters are modeled with reference to Faulhaber Motor K 3564 series parameters from their data sheet and presented as in the Appendix [18]. The required parameters of BLDC motor are taken as configurable parameters and modeled using state space representation of MATLAB model as shown in Figure 2.

### 2.2. Modeling of Inverter Circuit

BLDC Motor Driver is IGBT based inverter circuitry and operates with switching PWM signal as input and generates three-phase voltage to drive BLDC motor. The three hall sensors are placed at 120 electrical degrees in rotor and acquire the rotor position information as a feedback to the controller. Three hall sensors with eight combinations generate hall signals with 120-electrical-degree sensor phasing for six input combinations.  Table 1 represents Hall Signals representation of our proposed motor model with corresponding EMF signals [19].

**Figure 3:**
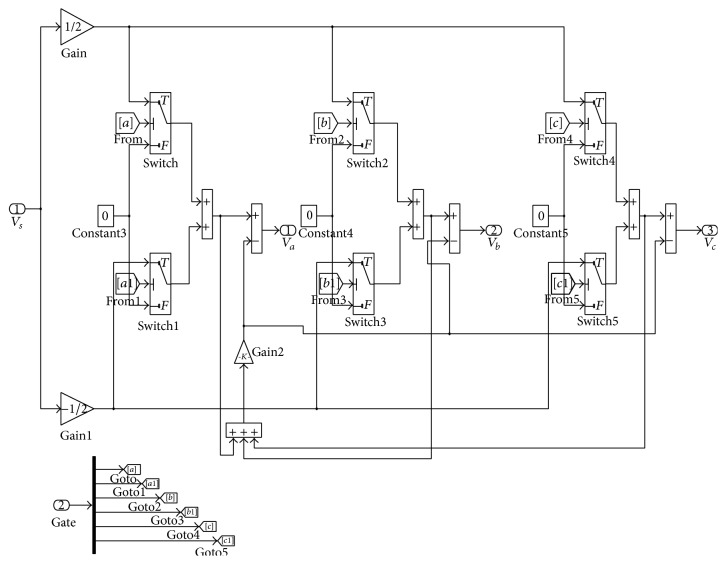
Inverter model for BLDC motor drive.

The inverter for BLDC motor drive is modeled using power electronics IGBT mathematical switches in MATLAB as shown in Figure 3. The PWM switching current control technique is implemented as shown in Figure 3 comparing input with relational operator to generate six PWM signals to drive inverter of BLDC motor.

### 2.3. Modeling of Encoder

**Figure 4:**
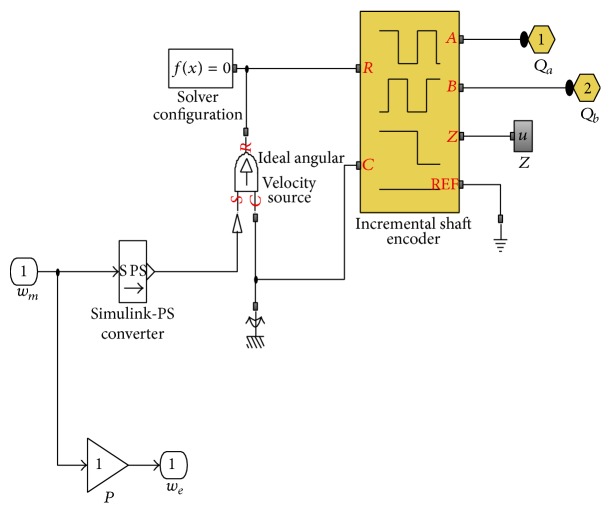
Encoder model for BLDC motor drive.

In modeling the control actuation system a quadrature encoder is implemented to acquire rotor position information from BLDC motor as a feedback to the controller. The shaft encoder model subsystem as shown in Figure 4 is attached to BLDC motor shaft. This encoder block will give the informative output of quadrature encoder pulses *Q*_*a*_ and *Q*_*b*_, which has velocity, direction, pulse frequency, and phase shifting position of rotor. The shaft encoder pulses are taken for computing the speed, position, and direction of motor. The entire computation is a triggered subsystem which is used to compute information of square wave coming from encoder using MATLAB Simscape model [20].

## 3. Modeling of Conventional PID Controller

The conventional Proportional-Integral-Derivative (PID) controllers are used in immense control actuation applications. The PID controller has the ability to eliminate steady-state error through integral action as the output changes corresponding to controller derivative action with respect to input command signal. The PID tuning method, namely, the Ziegler-Nichols method, confirms gain parameters needs to obtain the step response of the system [21].

The continuous control signal *u*(*t*) of the PID controller [22–24] is given by

(4) $$\begin{array}{c} u\left(\;t\;\right)=K_{p}e\left(\;t\;\right)+\left(\;\frac{1}{T_{i}}\;\right)\int e\left(\;t\;\right)dt+T\left(\;dt\;\right), \end{array}$$

where *K*_*p*_ is the proportional gain, *T*_*i*_ is the integral time constant, *T*_*d*_ is the derivative time constant, and *e*(*t*) is the error signal. The proportional, integral, and derivative terms enumerate to obtain desired output of the PID controller. *u*(*t*) as the output of PID-controller, equation to be the final form of the PID algorithm is

(5) $$\begin{array}{c} u\left(\;t\;\right)=K_{p}e\left(\;t\;\right)dt+K_{i}e\left(\;t\;\right)dt+K_{d}\frac{d}{dt}\cdot e\left(\;t\;\right), \end{array}$$

where *K*_*p*_ is proportional gain, a tuning parameter. *K*_*i*_ is integral gain, a tuning parameter. *K*_*d*_ is gain, a tuning parameter.

According to (5) control signals calculated for conventional PID gain parameters *K*_*p*_, *K*_*i*_, and *K*_*d*_.

## 4. Modeling of Fuzzy-PID Controller

**Figure 5:**
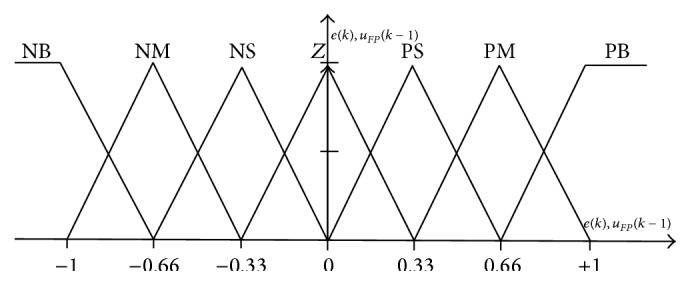
Membership functions for input and output variables.

In fuzzy-PID controller modeling a variable of drive module is selected as input and output. Here a speed error, *e*(*k*), and the delayed feedback control signal, *u*_*FP*_(*k* − 1), are considered as the inputs [24–26]. The output of the fuzzy-PID controllers is the gain, *FK*_*P*_. The fuzzy linguistic variable “*e*(*k*)” has ranges: positive large (PL), zero (ZE), and negative large (NL), with corresponding proportional membership function as shown in Figure 5. The fuzzy sets can be represented by universal limits −1, +1 *F*_*i*_, *i* = 1, 2, and *j* = 1, 2, 3. Their corresponding membership functions can be symbolized by *μF* ^*j*^(*e*, *u*_*FP*_(*k* − 1)), *j* = 1, 2.

The fuzzy controller is modeled using IF-THEN interference rules:

 
*R* ^(*k*)^: if *e*(*k*) is *F*_1_, and *u*_*F*_(*k* − 1) is *F* ^*l*^
 then *F* is *C*_*k*_^*l*^ for *j* = 1,…, 3, *l* = 1,…, 3, *k* = 1,…, 9.

The output from fuzzy-PID controller is derived from (3) and its output parameters are negative very large (NVL), negative large (NL), negative medium (NM), and negative small (NS) and zero, positive small (PS), positive medium (PM), positive large (PL), and positive very large (PVL). The same singleton membership functions of Figure 5 are used similar to the fuzzy sets and corresponding rule is formed in Table 2.

The final controller output is obtained from

(6) $$\begin{array}{c} FK_{P}=\alpha_{P}\left(\;e\left(\;k\;\right),u_{FP}\left(\;k-1\;\right)\;\right). \end{array}$$

For better control performance and simplicity structure a triangular shaped function is implemented as membership function with limits (−1, +1): a seven level is used for all input and output variables.

**Figure 6:**
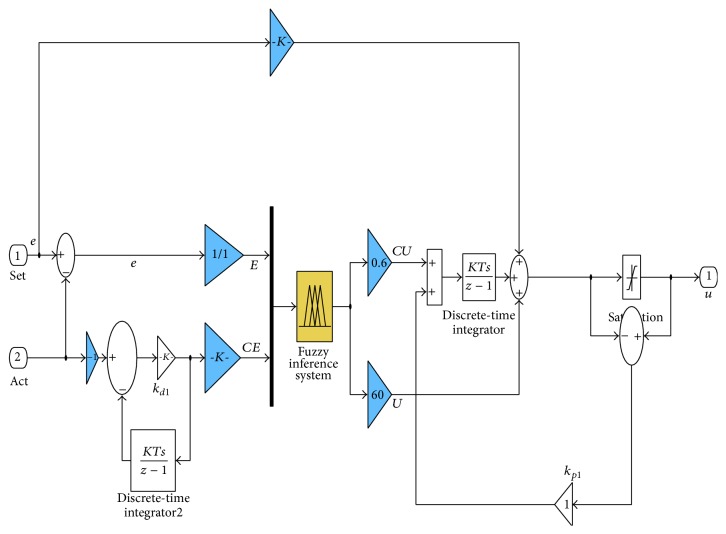
Fuzzy-PID controller for BLDC motor drive.

**Figure 7:**
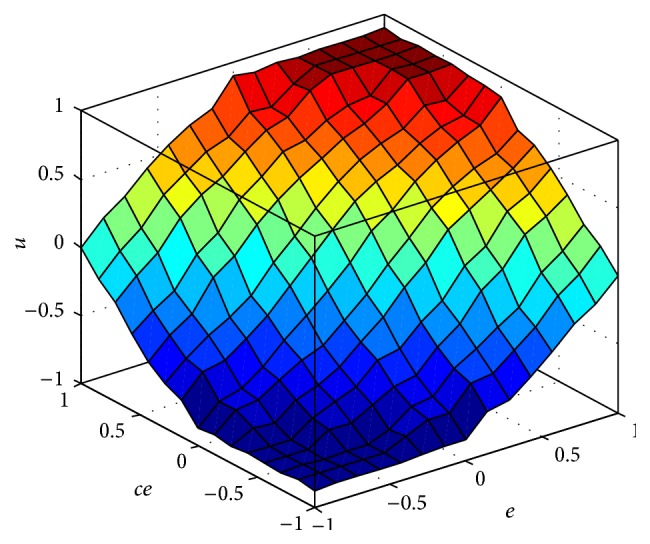
Surface viewer of fuzzy-PID controller for BLDC drive.

The fuzzy-PID controller is modeled in MATLAB/Simulink environment with gains *K*_*p*_, *K*_*i*_, and *K*_*d*_ which is shown in Figure 6 and its corresponding rule viewer in Figure 7 to analyze stability of the system.

## 5. Gearhead Modeling

The fins of missiles will be actuated from BLDC motor shaft drive through a planetary gearhead with gear ratio of 14 : 1. A planetary gear type has three wheels named Sun, Carrier, and Ring, in which the Ring and Carrier run anticlockwise with Sun gear [27].

The planetary gear and its ratio are calculated using equations below:

(7) ωs=21+sωc,ωr=1+2ssωc,

where *ω*_*s*_ is Angular Velocity of Sun gear. *ω*_*c*_ is Angular Velocity of Carrier gear. *ω*_*r*_ is Angular Velocity of Ring gear or Internal gear. *s* is gear ratio.

**Figure 8:**
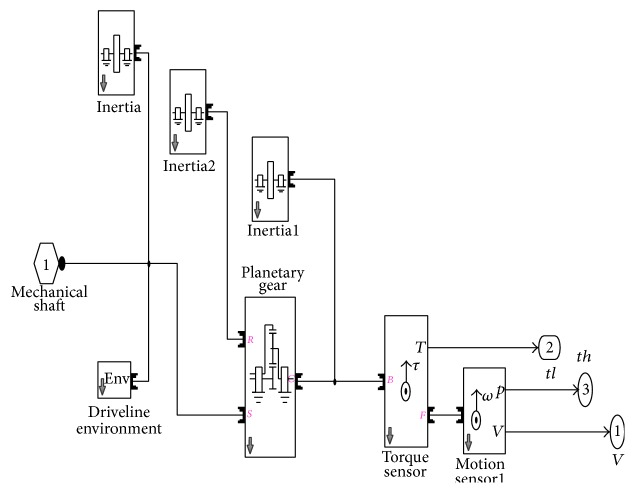
Gearhead modeling for control actuation system.

The simulation of drive train model is modeled using Simscape in which motor shaft is coupled with gearhead. This tool extends the fast capability in MATLAB/Simulink environment, in the future. The gearhead is modeled using SIM-DRIVELINE a tool of MATLAB with reduction ratio for Ring and Sun that is 14 : 1 as shown in Figure 8.

## 6. Results of Simulation

The complete actuation system is modeled in MATLAB to verify simulation studies of the proposed system. The MATLAB Simscape tools are used to model gear of motor and corresponding physical parameters. The results are confirmed by Faulhaber BLDC Motor K 3564 series specifications with 38(1/s) planetary gearheads that are referred to from supplementary materials I and II (in Supplementary Material available online at http://dx.doi.org/10.1155/2015/723298). An Incremental Decoder is modeled using Simscape Library Block to detect hall position sensor. The rotor position signals to generate gate signals as per Table 1 Hall Signals representation to corresponding gate signals switches of IGBT driver circuit. The trapezoidal back EMF is modeled as a function of rotor position from encoder using back EMF constant (*Ke*).

### 6.1. PID Controller

**Figure 9:**
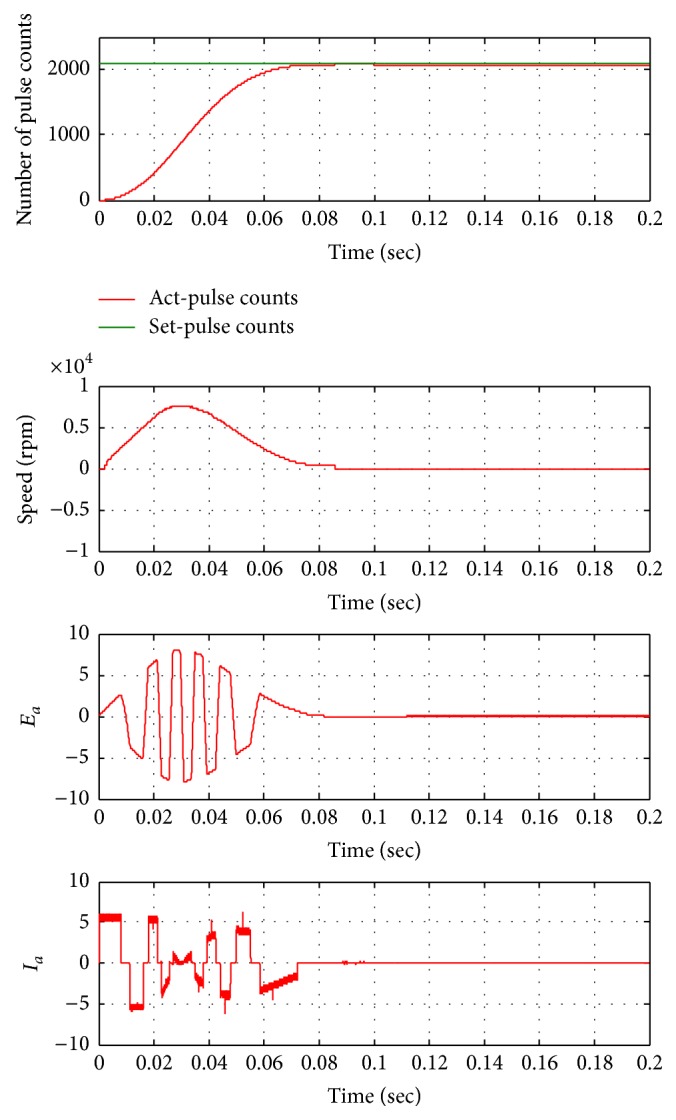
Position response of PID controller based control actuation system for 2100 counts.

The control actuation system using BLDC motor is modeled using PID controller. The system responses are obtained from various command signals. The PID controller based actuation system is tested for 2100 commands signals as shown in Figure 9. The PID controller for 2100 command signals has delay rise time (72.5 ms) which is critical for an actuation system. For different operating conditions of PID based control actuation system is unable to deliver better performance in nonlinear conditions for changing load conditions and different commanded signals. To compensate the demand of nonlinear controllers a fuzzy-PID is proposed.

### 6.2. Fuzzy-PID Controller

**Figure 10:**
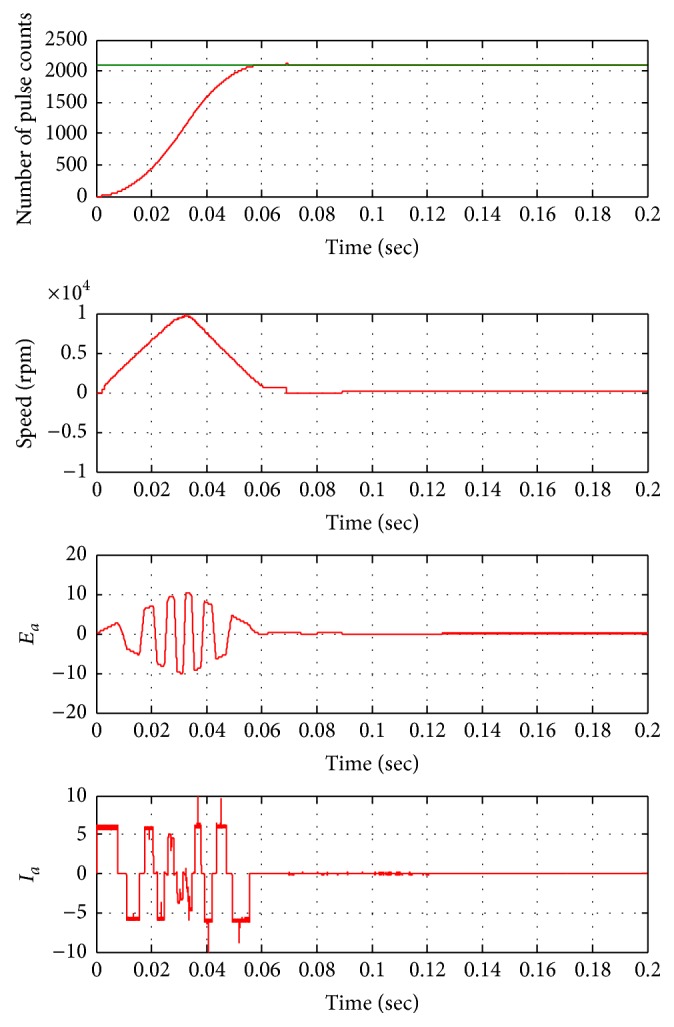
Position response of fuzzy-PID controller based control actuation system for 2100 counts.

**Figure 11:**
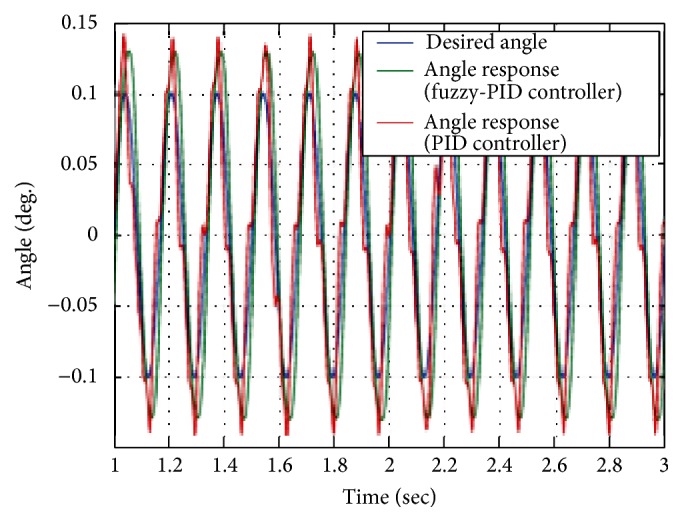
Angle response of PID and fuzzy-PID controller.

The control actuation system using BLDC motor is modeled using fuzzy-PID controller. Simulated BLDC motor parameters like speed, back EMF generated, and current of control actuation system are shown in Figure 10 for fuzzy-PID controller. Fuzzy PID controller reaches system load torque of 180 mN-m with operational time of 48 milliseconds. Fin control actuation angle analysis is carried out for the performance of conventional PID controller and fuzzy-PID controller. Figure 10 shows that fuzzy-PID controller has better performance than the conventional one for BLDC motor drive response. The drive fin control actuation system response for fuzzy-PID controller and PID controller is shown in Figure 11.

## 7. Hardware Results and Verification

**Figure 12:**
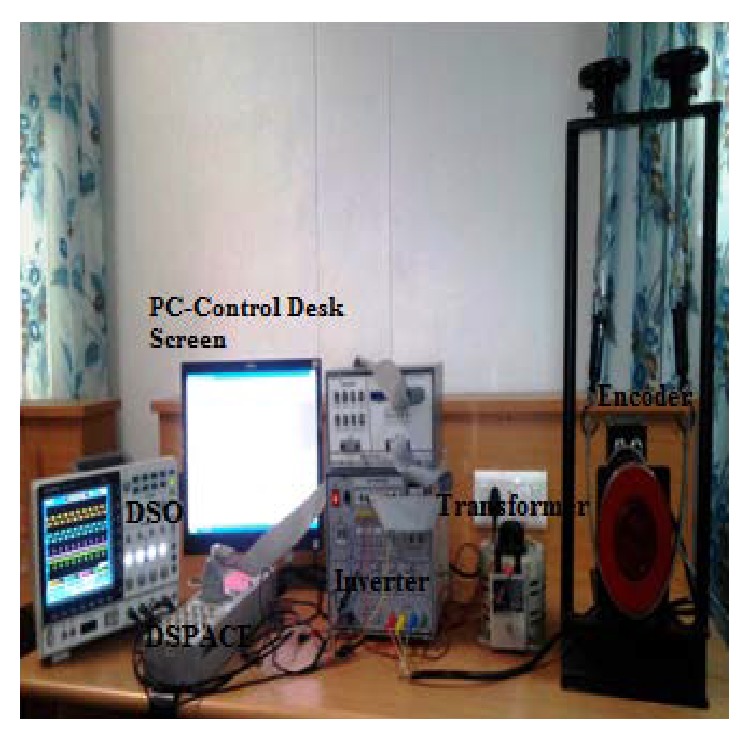
Hardware setup of control actuation system.

The Hardware results are verified for a proposed fuzzy-PID controller with DSPACE 1104 controller and a real time controller. The complete simulation model is simulated in MATLAB environment. Through control desk software MATLAB and dSPACE environment is interfaced to validate proposed systems stability for various load disturbances. The control actuation system using BLDC motor with encoder and gearhead is modeled, an interface with dSPACE controller test bench as shown in Figure 12.

**Figure 13:**
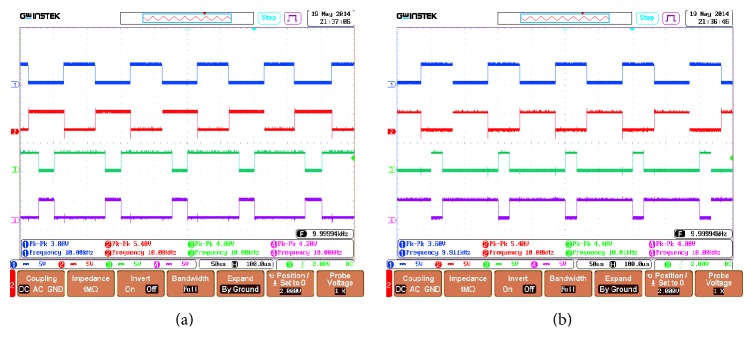
PWM duty cycle generation for fuzzy-PID controller signals.

Figures 13(a) and 13(b) show the PWMs generated by the dSPACE controller which is given as input to drive the IGBT switches with the switching frequency of 10 KHz. Separate encoder has been modeled for 100 ppr. From the QEP signals *Q*_*a*_ and *Q*_*b*_, speed and position of motor shaft are calculated using pulse count decoder block. System builds with inner loop as current control and outer loop as speed/position control as shown in Figure 15. Current *I*_dc_ is controlled through fixed PWM of 10 KHz and it is limited from 6 A peak to −6 A peak. Necessary hall signal decoding logic is built in with the proposed system. Feedback controller for both current and speed/position was developed with conventional PID as well as fuzzy-PID controller. A motion controller is designed using fuzzy-PID control as input is set as step input for 2100 counts.

### 7.1. Speed Control Performance

**Figure 14:**
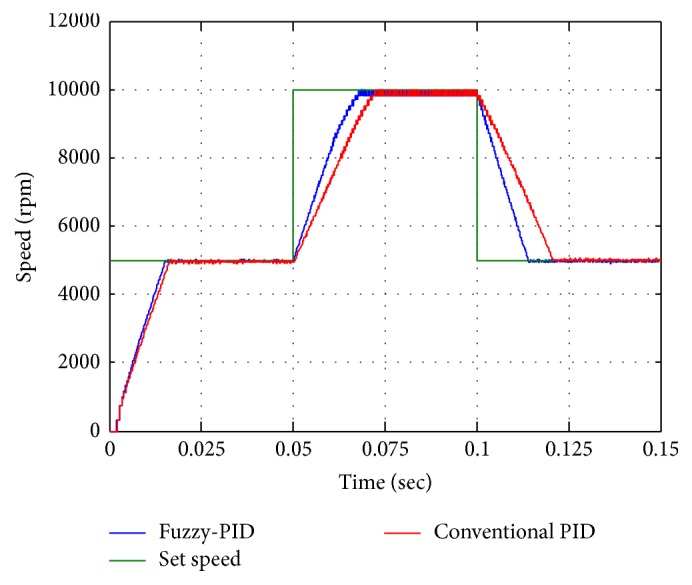
Step speed change (5000–10000–5000) rpm.

The result shows the speed control of motor with inner loop current control for both conventional PID and fuzzy-PID (see Figure 14). The closed loop speed with 10000 rpm of set speeds both conventional PID starting response and fuzzy-PID starting response as shown in Figure 14. Fuzzy-PID controller performance has better rise time with minimum peak to peak overshoot for the desired speed.

**Figure 15:**
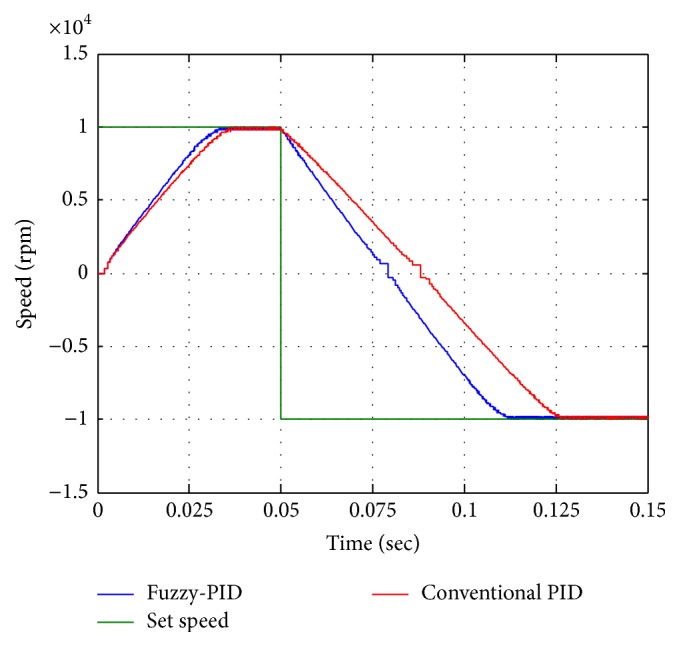
Speed reversal performances.

For step change of speed analysis is as shown in Figure 15 the speed step rose from 5000 rpm into 10000 rpm at 0.05 sec then step down from 10000 rpm into 5000 rpm at 0.1 sec. Analysis suggests that fuzzy performance is better than conventional controller in considering the settling errors. From the step change analysis fuzzy-PID improves the performance of the actuation system compared with conventional PID.

### 7.2. Position Control Performance

**Figure 16:**
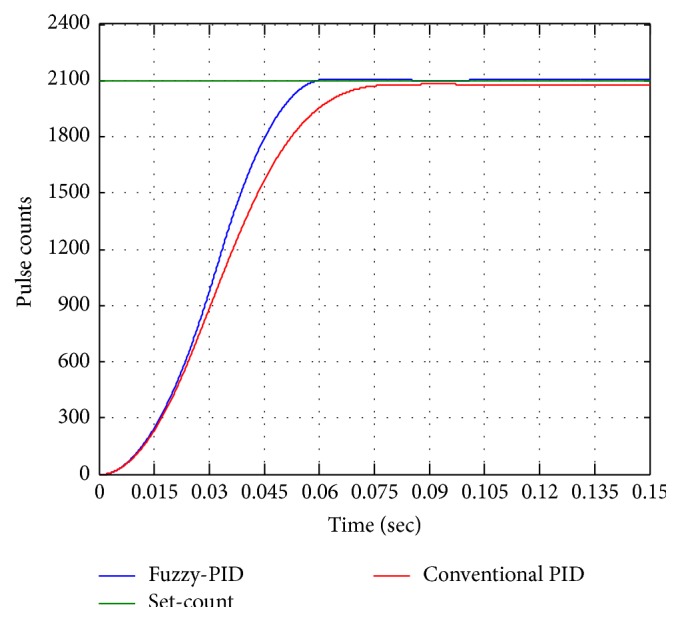
Position control at 2100 pulse counts.

The position control step analysis is carried out for control actuation system.  Figure 16 shows the position control equivalence pulse counts of BLDC motor with inner loop current control for both conventional PID and fuzzy-PID. Unit step change of fins movement for 1 degree required is 2100 pulse counts from motor shaft, with step analysis as reference for desired 2100 pulse counts. The step responses of conventional PID and fuzzy-PID are shown in Figure 16. From the experimental results it is observed that conventional PID requires 75 ms to settle with 1.15% of error. Fuzzy-PID requires 52.5 ms to reach the set target with 0.01% of error.

**Figure 17:**
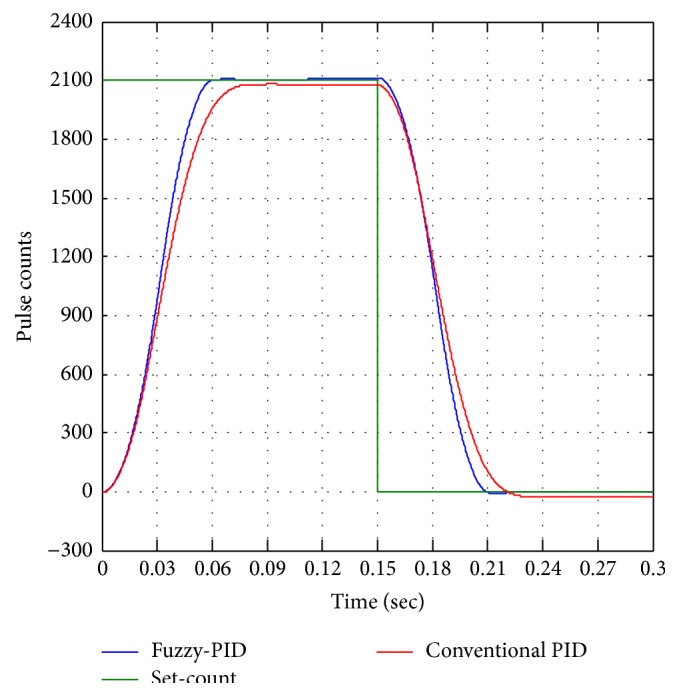
Step change from 2100 to 0 pulse counts.

**Figure 18:**
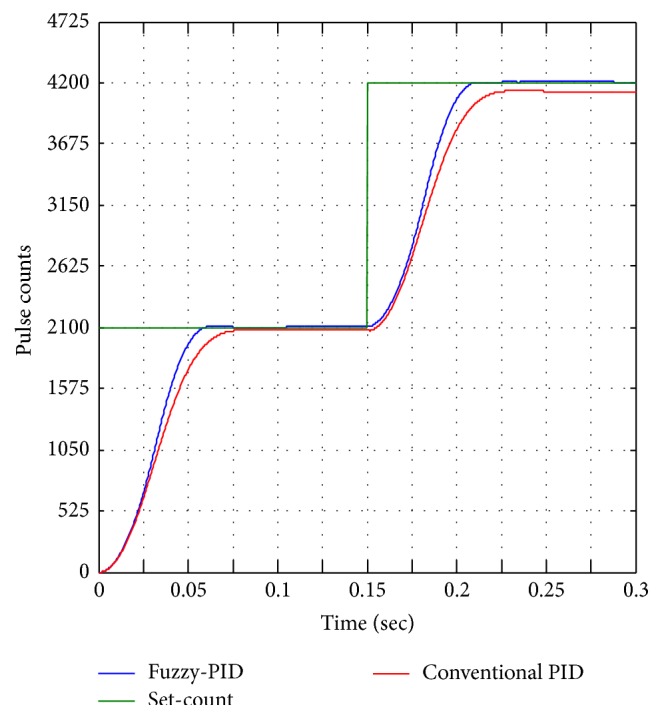
Step change from 2100 to 4200 pulse counts.

Further analyses of step change position control are shown in Figure 17, step change from pulse counts of 2100 into 0 pulse counts. The BLDC motor operates in reverse to reach the initial pulse count (0), and from the experimental results fin moves forward and reverse (to reach 0 pulse counts) and required the same time to reach unit set-pulse counts 50 for both conventional and fuzzy-PID controllers.  Figure 18 shows the step increment analysis of unit step change of fin from 1-degree to 2-degree equivalent pulse counts of 2100 to 4200 pulse counts. From the various step analyses fuzzy performance is better than conventional controller with detailed step performance parameter of rise time, peak overshoot, and percentage error as presented in Table 3.

## 8. Conclusion

A fuzzy-PID based control actuation system has been proposed with planetary gearhead modeling. A self-tuning with optimum gain parameter of fuzzy controller has been employed to control BLDC motor drive. The position of fins is controlled by controlling BLDC motor input voltage using gains values. An electronic commutation of BLDC motor is used from the error between input and reference commanded values. Moreover, a quadrature type encoder is used to sense accurate rotor position information and as a feedback controller. An improved dynamic response of BLDC motor drive has been achieved for a wide range of step response commanded values. Experimental results have shown excellent performance of proposed fuzzy-PID controller and are well demonstrated for uncertain nonlinear conditions. The rise time of BLDC motor drive for fuzzy-PID controller is well within 52.5 (ms). In this way, performance of fuzzy technique attracts engineers and practitioners to develop fuzzy based control actuation system in fins and glider application.

## Supplementary Material

Supplementary table 1: The primers used as reference motor parameters for modeling Brushless DC motor in MATLAB SIMULINK as per faulhaber motor data sheet listed in this table.
Supplementary table 2: The primers used as reference shaft encoder parameters for modeling planetary in MATLAB-SIMSCAPE as per faulhaber encoder data sheet listed in table.

## Figures and Tables

**Table 1:** Hall and back EMF signals.

ha	hb	Hb	EMF_*a*_	EMF_*b*_	EMF_*c*_
0	0	0	0	0	0
0	0	1	0	−1	+1
0	1	0	−1	+1	0
0	1	1	−1	0	+1
0	0	0	+1	0	−1
0	0	1	+1	−1	0
0	1	0	0	+1	−1
0	1	1	0	0	0

**Table 2:** 7 × 7 rule base table for fuzzy-PID controller.

*e*, *u*_*FP*_(*k* − 1)	NB	NM	NS	ZO	PS	PS	PB
NB	NB	NB	NB	NB	NM	NS	ZO
NM	NB	NB	NB	NM	NS	ZO	PS
NS	NB	NB	NM	NS	ZO	PS	PM
ZO	NB	NM	NS	ZO	PS	PM	PB
PS	NM	NS	ZO	PS	PM	PB	PB
PM	NS	ZO	PS	PM	PB	PB	PB
PB	ZO	PS	PM	PB	PB	PB	PB

**Table 3:** Results of PID controller based control actuation system.

Parameters	Fuzzy PID	Conventional PID
Rise time—*T*_*r*_ (ms)	52.5	72.5
Settling time—*T*_*s*_ (ms)	56.5	77.8
Deceleration time—*T*_*d*_ (ms)	50.24	5.3
Transient behavior	Smooth	Oscillatory
Peak overshoot	2.7	13
% error	0.01	1.15

**Table 4:** Specifications of BLDC motor.

S. number	Parameters	Values
1	Nominal voltage	24 volts
2	Terminal resistance (phase to phase)	1.16 Ohm
3	Output power	101 W
4	Back EMF constant	2.107 mV/rpm
5	Torque constant	20.12 mNm/A
6	Rotor inertia	34 gcm^2^
7	Gearhead type	30/1 s

## Appendix

See Table 4.
