# Supplementary material for: Modeling and Simulation of Control Actuation System with Fuzzy-PID Logic Controlled Brushless Motor Drives for Missiles Glider Applications
Source: ScientificWorldJournal. 2015 Nov 3;2015:723298. doi: 10.1155/2015/723298 (PMC4647030; doi:10.1155/2015/723298)
Supplement: Supplementary file 1 — Supplementary table 1: The primers used as reference motor parameters for modeling Brushless DC motor in MATLAB SIMULINK as per faulhaber motor data sheet listed in this table. Supplementary table 2: The primers used as reference shaft encoder parameters for modeling planetary in MATLAB-SIMSCAPE as per faulhaber encoder data sheet listed in table. [file 723298.f1.pdf]

Brushless DC-Servomotors

47,1 mNm

For combination with
Gearheads:
30/1(S), 32/3(S), 38A, 38/1(S), 38/2(S)
Encoders:
IE3-1024(L), 5500, 5540
Drive Electronics:
Speed Controller, Motion Controller

Series 3564 ... B

	3564 K			012 B	024 B	036 B	048 B	
1	Nominal voltage	U_N		12	24	36	48	Volt
2	Terminal resistance, phase-phase	R		0,6	1,2	2,8	4,4	Ω
3	Output power [1]	$P_{2\ max.}$		109	101	101	101	W
4	Efficiency	$\eta_{max.}$		81	81	81	82	%
5	No-load speed	n_o		7 850	11 300	11 550	12 200	rpm
6	No-load current (with shaft ø 4,0 mm)	I_o		0,206	0,189	0,131	0,109	A
7	Stall torque	M_H		291	371	379	401	mNm
8	Friction torque, static	C_o		1,10	1,10	1,10	1,10	mNm
9	Friction torque, dynamic	C_v		$2,4 \cdot 10^{-4}$	$2,4 \cdot 10^{-4}$	$2,4 \cdot 10^{-4}$	$2,4 \cdot 10^{-4}$	mNm/rpm
10	Speed constant	k_n		658	475	324	258	rpm/V
11	Back-EMF constant	k_E		1,521	2,107	3,089	3,877	mV/rpm
12	Torque constant	k_M		14,52	20,12	29,50	37,02	mNm/A
13	Current constant	k_I		0,069	0,050	0,034	0,027	A/mNm
14	Slope of n-M curve	$\Delta n/\Delta M$		27	31	31	31	rpm/mNm
15	Terminal inductance, phase-phase	L		96	194	427	678	µH
16	Mechanical time constant	τ_m		10	11	11	11	ms
17	Rotor inertia	J		34	34	34	34	gcm^2
18	Angular acceleration	$\alpha_{max.}$		86	109	111	118	$\cdot 10^3 rad/s^2$
19	Thermal resistance	$R_{th\ 1}$ / $R_{th\ 2}$	2,5 / 6,3					K/W
20	Thermal time constant	τ_{w1} / τ_{w2}	23 / 1 175					s
21	Operating temperature range		– 30 ... +125					°C
22	Shaft bearings		ball bearings, preloaded					
23	Shaft load max.:							
	– radial at 3 000/20 000 rpm (7,4 mm from mounting flange)		108 / 73					N
	– axial at 3 000/20 000 rpm (push-on only)		50 / 30					N
	– axial at standstill (push-on only)		131					N
24	Shaft play:							
	– radial	≤	0,015					mm
	– axial	=	0					mm
25	Housing material		aluminium, black anodized					
26	Weight		310					g
27	Direction of rotation		electronically reversible					
**Recommended values** - mathematically independent of each other								
28	Speed up to [2]	$n_{e\ max.}$		27 000	27 000	27 000	27 000	rpm
29	Torque up to [1] [2]	$M_{e\ max.}$		47,1	44,0	43,9	44,0	mNm
30	Current up to [1] [2]	$I_{e\ max.}$		3,68	2,50	1,71	1,36	A

[1] at 22 000 rpm
[2] thermal resistance $R_{th\ 2}$ by 55% reduced

Note:
The diagram indicates the recommended speed in relation to the available torque at the output shaft for a given ambient temperature of 22°C.

The diagram shows the motor in a completely insulated as well as thermally coupled condition ($R_{th\ 2}$ 55% reduced).

The nominal voltage (U_N) curve shows the operating point at nominal voltage in the insulated and thermally coupled condition. Any points of operation above the curve at nominal voltage will require a higher operating voltage. Any points below the nominal voltage curve will require less voltage.

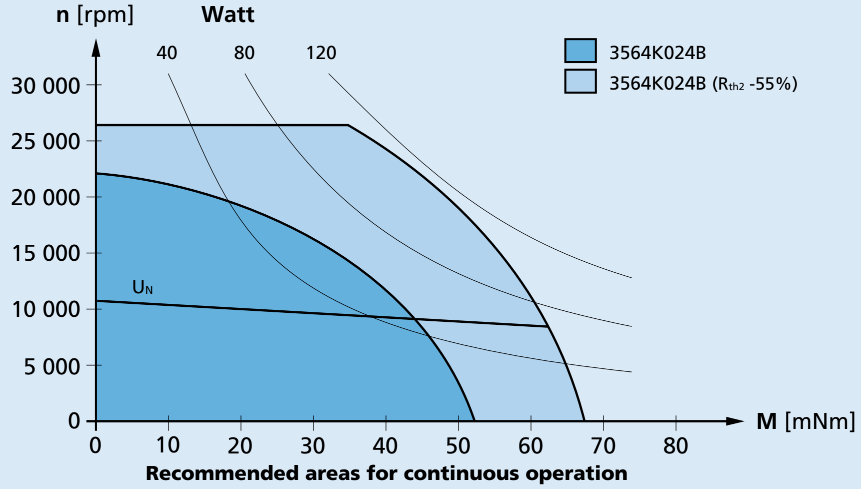

Recommended areas for continuous operation

© DR. FRITZ FAULHABER GMBH & CO. KG
Specifications subject to change without notice.
www.faulhaber.com

Options
K1000:
Motors in autoclavable version.
K1155:
Motors for operation with Motion Controllers

3564 K ... B

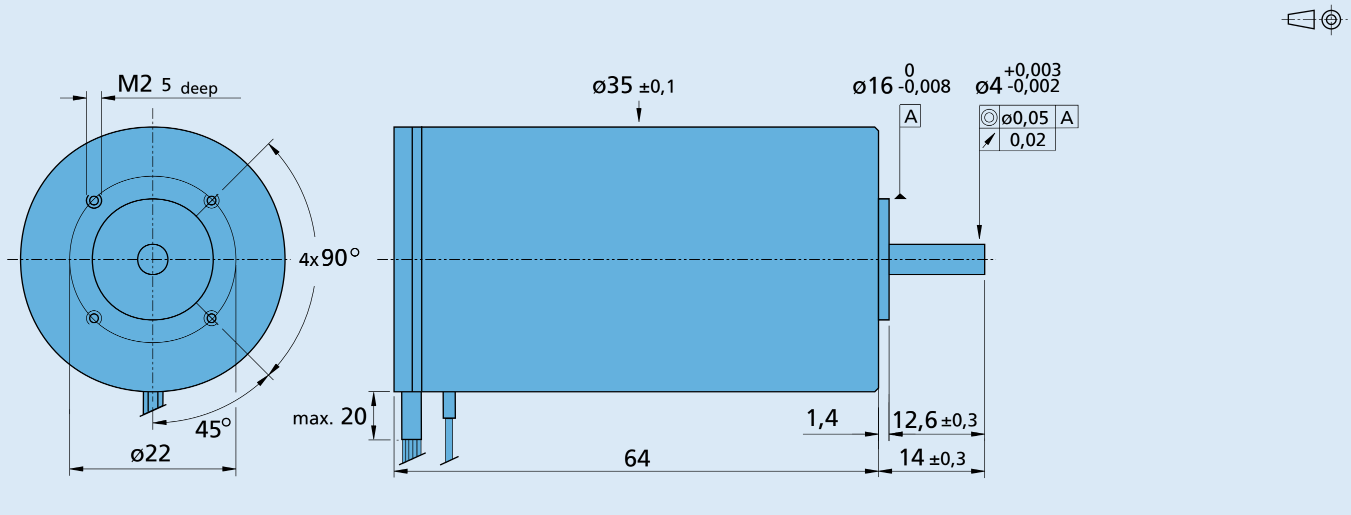

3564 K ... B - K312 with rear end shaft

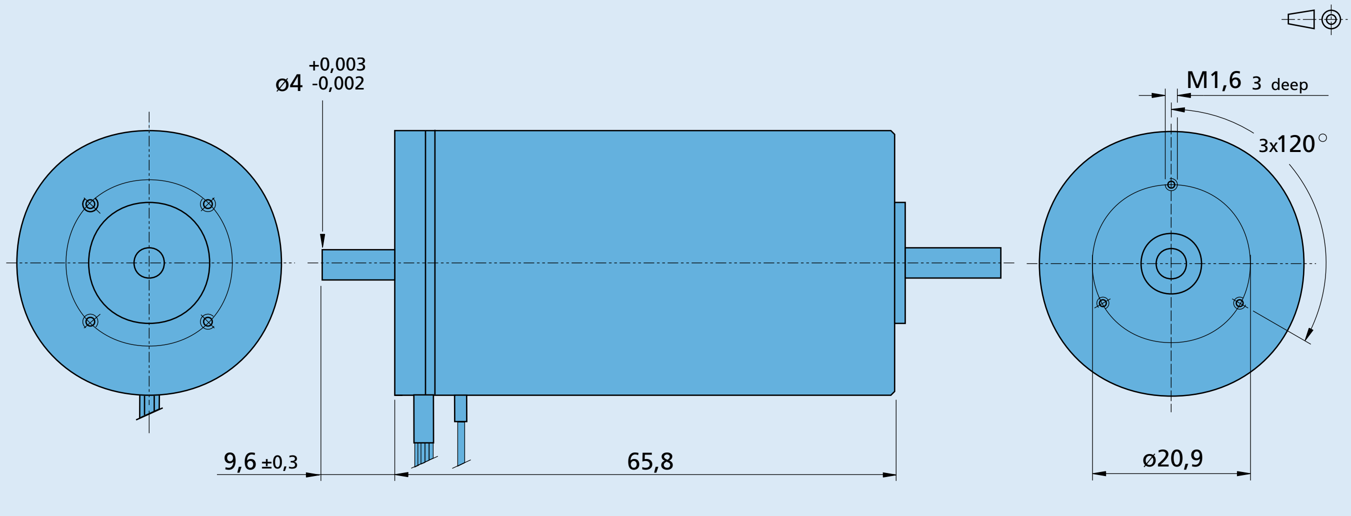

Cable and connection information

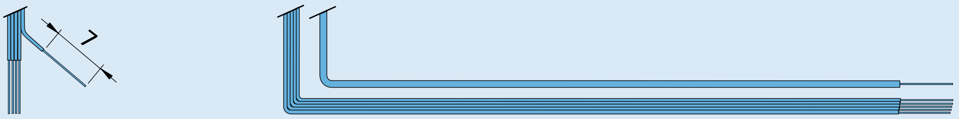

Cable
Single wires, material PTFE
Length 300 mm ± 15 mm
3 conductors, AWG 20
5 conductors, AWG 26

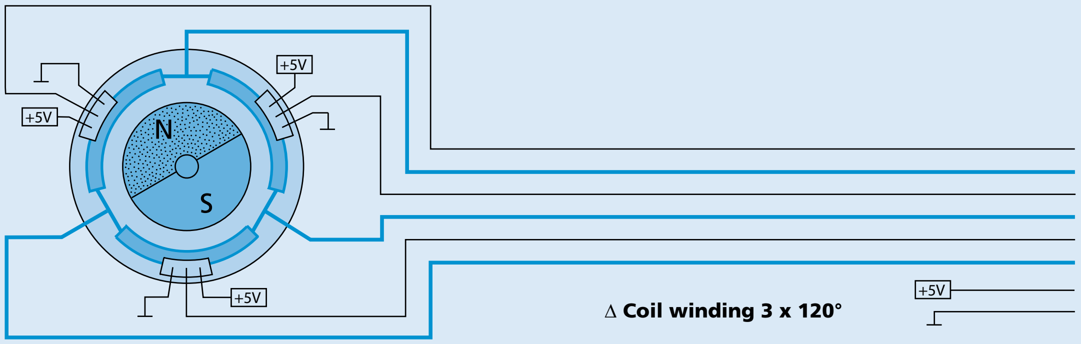

Connection

	Function	Colour
A	Hall sensor	green
A	Phase	brown
B	Hall sensor	blue
B	Phase	orange
C	Hall sensor	grey
C	Phase	yellow
+5V	Logical supply	red
GND	Logical	black

© DR. FRITZ FAULHABER GMBH & CO. KG
Specifications subject to change without notice.
www.faulhaber.com
